# Uterine Rupture with Massive Late Postpartum Hemorrhage due to Placenta Percreta Left Partially In Situ

**DOI:** 10.1155/2013/906351

**Published:** 2013-12-10

**Authors:** Mehmet Coskun Salman, Pinar Calis, Ozgur Deren

**Affiliations:** Department of Obstetrics and Gynecology, Hacettepe University Faculty of Medicine, Sihhiye, 06100 Ankara, Turkey

## Abstract

Placental adhesive disorders involve the growth of placental tissue into or through the uterine wall. Among these disorders, placenta percreta is the rarest one. However, it may cause significant complications. This report aimed to report a neglected patient with placenta percreta who developed uterine rupture with life-threatening late postpartum intra-abdominal hemorrhage. On admission, the patient had acute abdomen with moderate abdominal distention and was subjected to emergency laparotomy. A full-thickness defect of the anterior uterine wall involving the hysterotomy site was seen. Placental tissues occupied both sides of the incision and posterior bladder wall was also invaded by placenta. Total abdominal hysterectomy with partial resection of the posterior bladder wall was performed.

## 1. Introduction

Placental adhesive disorders involve the growth of placental tissue into or through the uterine wall. Among these disorders, placenta accreta is the most common type where the chorionic villi are in contact with myometrium. The rarest one is placenta percreta in which the chorionic villi invade through uterine serosa and may involve the adjacent organs [1]. Therefore, placenta percreta may cause significant complications due to massive hemorrhage, infection, and injury to surrounding organs. Also, placenta percreta was reported to be associated with maternal mortality in approximately 6% of cases [2]. The management of patients with placenta percreta is of vital importance accordingly. Traditional management consisted of cesarean hysterectomy that may be associated with significant intraoperative and postoperative complications [3]. However, optimal management has yet to be defined and conservative management options are becoming more widely accepted to avoid surgery-related morbidity and even mortality [4]. Nevertheless, conservative approaches are not devoid of serious complications [4, 5].

The aim of this report is to describe a neglected case of placenta percreta initially managed conservatively who developed uterine rupture with life-threatening late postpartum intra-abdominal hemorrhage. The management options for placenta percreta were discussed as well.

## 2. Case Presentation

**Figure 1:**
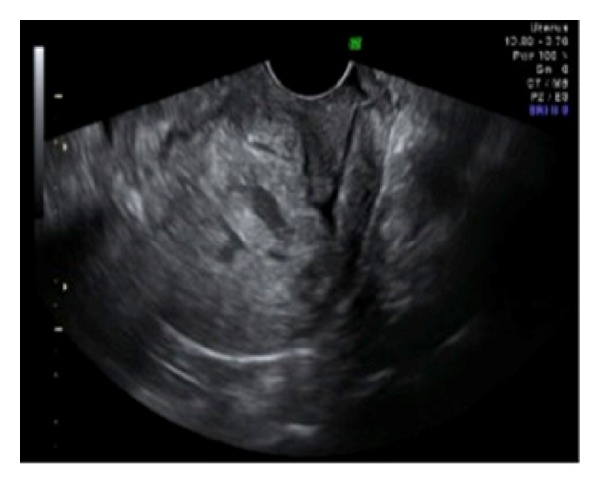
Ultrasonography showing soft tissue density with irregular contour in the endometrial cavity.

**Figure 2:**
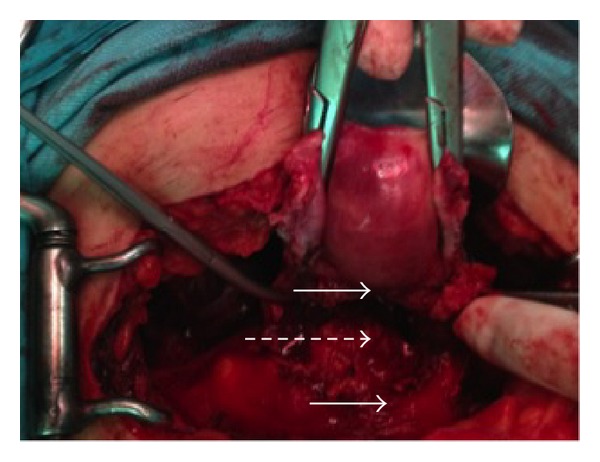
Anterior uterine wall defect on hysterotomy incision (solid arrows show the lower and upper borders of the defect and dashed arrow shows exposed posterior uterine wall).

**Figure 3:**
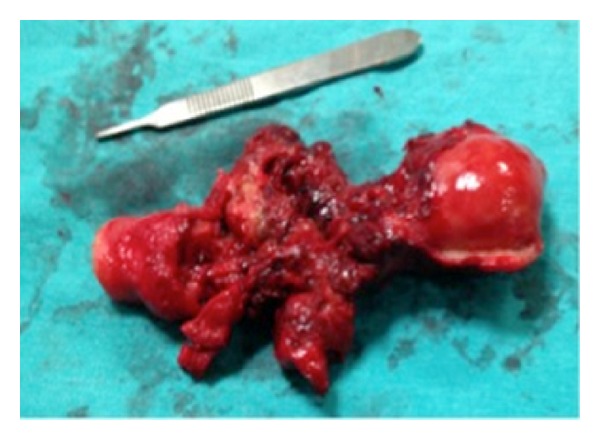
Total abdominal hysterectomy specimen.

A 35-year-old gravida 3, parity 2 woman was admitted to the hospital for vaginal bleeding and severe abdominal pain. Her past medical history was free of any medical problems. On her obstetric history, she had a vaginal delivery following an uneventful pregnancy course 8 years ago. Her second pregnancy was terminated via hysterotomy at 18th weeks due to multiple fetal abnormalities including hydrocephalus. She was diagnosed to have total anterior placenta previa on her third pregnancy. This last pregnancy was otherwise uneventful and the patient gave birth to a term, healthy infant via planned repeat cesarean section in a private hospital. During the operation, partially adherent placenta had been detected and the adherent part of the placenta had been left in situ. Postoperative treatment had not been given. 20 days after the operation, she had been hospitalized due to massive vaginal bleeding and had been transfused four units of packed red blood cells since her blood count revealed severe anemia with a hemoglobin level of 5.6 gr/dL. She had been discharged from hospital three days later. However, one week later, she had presented to another center due to vaginal bleeding and pelvic pain. She had been referred to our hospital with a presumptive diagnosis of placental retention because ultrasonography showed soft tissue density with irregular contour in the endometrial cavity (Figure 1). On her admission, she had findings consistent with acute abdomen and moderate abdominal distention was detected. Her blood pressure was 90/60 mmHg and heart rate was 110/minute. Hemoglobin was 8.5 gr/dL on complete blood count. Ultrasonography revealed free fluid filling the pelvis and lower abdomen. Her serum human chorionic gonadotropin was negative. After obtaining informed consent including hysterectomy, an emergency laparotomy was performed. Following drainage of approximately 1000 mL of free blood from the abdominal cavity, a full-thickness defect on the anterior wall of the uterus was seen (Figure 2). The defect involved the hysterotomy incision and both sides of the incision were occupied by irregular placental tissue with no obvious normal myometrium. Blood was oozing from the defect. Posterior bladder wall was also invaded by placenta and mobilization of the bladder off the lower uterine segment could not be achieved. Total abdominal hysterectomy with partial resection of the posterior bladder wall was performed (Figure 3). The patient was discharged from the hospital on fourth postoperative day with a bladder catheter that was removed one week after the operation. She was asymptomatic on her control visit performed six weeks later.

## 3. Discussion

Placenta percreta is among the most devastating pregnancy complications since it may be associated with significant maternal morbidity and even mortality [2]. Majority of the cases have a history of prior cesarean section and placenta previa as the main risk factors and the incidence has gradually increased in the last decades mostly due to the increased utilization of cesarean deliveries [1, 6]. Therefore, pregnant women with such risk factors should be evaluated antenatally for placental adhesive disorders to be able to get ready for the management options.

The management of placenta percreta is a challenge for the obstetrician because optimal management has not been defined clearly so far and there is no treatment approach that is obviously without risk [7]. Although the traditional management includes immediate definitive surgery consisting of cesarean hysterectomy, it may be associated with significant blood loss and injury to urinary tract with extended and complicated postoperative course. Also, massive transfusion and sometimes partial cystectomy may be required during such an approach [3]. According to a review of the literature, when hysterectomy is performed at the time of delivery, morbidity includes urologic complications in 72%–81% of cases and partial cystectomy in 40% [2]. In cases with antenatal diagnosis, planned preterm cesarean hysterectomy with the placenta left in situ may be performed following the transfer of patient to a tertiary perinatal care center [8]. Thus, extreme blood losses and neighboring organ injuries may be avoided [9, 10]. However, this management is possible only with antenatal diagnostic confirmation which may be achieved effectively by gray scale ultrasound [8]. On the other hand, diagnosis may be difficult in less severe cases and in cases without risk factors. Furthermore, increasing incidence trends and desire for future fertility warrants more conservative management options. Especially in cases with percreta invading adjacent organs, mortality and morbidity may be reduced when the patient is managed conservatively [11].

Conservative management of adhesive placenta classically involves a fundal or posterior uterine incision to avoid placenta. Following the delivery of the infant, the uterine incision is closed with the placenta left in situ [11]. Other conservative treatment options reported in the literature include embolization of the uterine arteries, methotrexate therapy, and uterus preserving surgeries including hemostatic sutures and arterial ligation with no superiority for one over the other [12]. In some cases, more than one conservative approach may be preferred in combination. However, conservative management may lead to severe complications including late postpartum hemorrhage, infection, sepsis, coagulopathy, emergent hysterectomy, and recurrence of placental adhesive disorders [11, 12]. According to a recent large literature review, conservative management of adhesive placenta is associated with secondary hysterectomy in 6%–31% of cases and maternal mortality in 0.3%–4%. Also, severe vaginal bleeding should be expected in 25%–53% of patients [12].

In the current case, an antenatal diagnosis of invasive placentation could not be made despite the fact that she was at high risk for invasive placentation. Therefore, adherent placenta had been detected unexpectedly during the repeat cesarean delivery and the placenta had been left partially in situ at the incision site although the patient did not have a desire for future fertility. Postoperatively, no additional therapeutic options were offered. The patient experienced massive vaginal bleeding approximately 3 weeks after the delivery. At that time, she was transfused without any further diagnostic evaluation because her serum chorionic gonadotropin (hCG) was in normal limits. Nevertheless, normal hCG levels may be detected without placental involution in conservatively managed patients [7]. One week later, the patient experienced recurrent bleeding with severe pelvic pain and was diagnosed to have abnormal imaging findings. Thereafter she was transferred to our center where a decision to proceed with emergency laparotomy was made. During the operation, uterine rupture was seen at the incision site where no healthy myometrial tissue was present. The rupture probably resulted from the necrosis of partially left placental tissue that was vascularized poorly. Bleeding from the ruptured area was substantial with apparent hemoperitoneum that necessitated hysterectomy with partial cystectomy without any other intraoperative or postoperative complications.

In conclusion, placental adhesive disorders should be searched thoroughly during antenatal visits in patients with risk factors. Following diagnostic confirmation, planned preterm cesarean hysterectomy with the placenta left in situ may be performed in cases with no desire for future fertility. When fertility is desired, the patient may be subjected to conservative management, but the patient should be informed about the possibility of significant risks and regular visits with short intervals should be undertaken. Additional interventions including methotrexate and interventional radiologic approaches may be considered.
